# On Dyspepsia; with Remarks, Submitted in Support of the Opinion That the Proximate Cause of This and of All Other Diseases Affecting the General System Is Vitiation of the Blood

**Published:** 1847-10

**Authors:** 


### ART. XXI.

*On Dyspepsia; with Remarks, submitted in support of the Opinion that the Proximate Cause of this and of all other Diseases affecting the General System is Vitiation of the Blood.* By JOHN BURDETT STEWARD, M.D., Fellow of the Royal College of Physicians.—*London,* 1847. 8vo, pp. 106.

DR. STEWARD "admits his conviction to be, that the proximate cause of all diseases consists in some alteration in the force, quantity, or quality of the circulating fluid; and that of those affecting the general system, vitiation of the blood is an invariable accompaniment." This principle is that which runs through his 'Remarks.'

On looking to see how Dr. Steward explains the origin of *indigestion,* and shows its proximate cause to be some change in the blood, we find him turning the tables on himself, and showing how indigestion itself is the cause of the change.

"A person, at the time healthy, either from quantity or quality of the food or fluid taken, or from grief, or any other cause equal to produce it, has an attack of what is called indigestion, that is to say, the food does not undergo the required changes; weight, uneasiness, and nausea are present; the concoction is imperfect, and the chyme necessarily suffers in character from this imperfect concoction, being less fit to form the composition which it is destined to become by the action of the bile. In other words, the chyme . . . . thus altered, when acted upon in the duodenum by the bile, must produce a chyle also less than perfect. . . . . The blood, therefore, with which this chyle, so changed, becomes mixed, must suffer deterioration in an equal degree." (p. 32.)

Here we have functional derangement, or disease of the digestive organs, followed by imperfect "concoction," and the blood is allowed to be healthy: what then becomes of Dr. Steward's conviction? How can he explain the action of grief upon the digestive organs, unless, indeed, he allows that the proximate cause of indigestion may consist in some change in the nervous system? It does not suit Dr. Steward's views to inquire into changes of this kind, for the more he inquires into them, the more he is bewildered—the deeper he gets into "mystery." It might have occurred to Dr. Steward that some of these difficulties, and the discovery of these unfathomable "mysteries," may be as fairly attributable to the mind of the individual inquiring, as to the subject itself. So appalled is Dr. Steward by the initiatory studies requisite for a comprehension of the physiology of the nervous system, and of the relations of that system to morbific changes in structure and function, and even to changes in the blood itself, that he will not enter upon them at all; and, in short, rejects all consideration of the nervous system whatever.

"It is not because I think lightly of its influence, that I reject reference to the nervous system, but because, in referring to it, every step we take leads us further and further into mystery. Not only is the power by which it works its effects hidden from us, but a knowledge of its changes during life being impossible, and after death doubtful, our reasoning must be hypothetical, and our treatment therefore uncertain." (p. 16.)

We need scarcely subjoin to this extract that Dr. Steward adds nothing to the pathology of the blood; gives no new researches showing its changes during life or after death, and, of course, tells his readers nothing about the changes it experiences in dyspepsia, except those hypothetical inferences which any one of his readers, whether medical or non-medical (for the book is addressed to both classes), might not as readily make as Dr. Steward himself.

With regard to the treatment of dyspepsia advocated by Dr. Steward, we need only add that it is the same as adopted by nine tenths of the practitioners of the United Kingdom. The book is, in short, a good manual for Dr. Steward's friends and patients; to them he can properly address Abernethy's counsel—"read my book," and to them it will be useful, and for them, we are inclined to think, it was written, and not for us, or for the literary and philosophical practitioner.

